# Rural training pathways: the return rate of doctors to work in the same region as their basic medical training

**DOI:** 10.1186/s12960-018-0323-7

**Published:** 2018-10-22

**Authors:** Matthew R. McGrail, Belinda G. O’Sullivan, Deborah J. Russell

**Affiliations:** 1 University of Queensland, Rural Clinical School, 78 on Canning Street, Rockhampton, QLD 4700 Australia; 2 Monash Rural Health, Monash University, 26 Mercy Street, Bendigo, VIC 3550 Australia; 3 Flinders University, Northern Territory, PO Box 41326, Casuarina, NT 0815 Australia

**Keywords:** Workforce, Rural training, Retention, Education, Recruitment, Location, Rural pathways

## Abstract

**Background:**

Limited evidence exists about the extent to which doctors are returning to rural region(s) where they had previously trained. This study aims to investigate the rate at which medical students who have trained for 12 months or more in a rural region return to practice in that same region in their early medical career. A secondary aim is to investigate whether there is an independent or additional association with the effect of longer duration of rural exposure in a region (18–24 months) and for those completing both schooling and training in the same rural region.

**Methods:**

The outcome was rural region of work, based on postcode of work location in 2017 for graduates spanning 1–9 years post-graduation, for one large medical program in Victoria, Australia. Region of rural training, combined with region of secondary schooling and duration of rural training, was explored for its association with region of practice. A multinomial logistic regression model, accounting for other covariates, measured the strength of association with practising in the same rural region as where they had trained.

**Results:**

Overall, 357/2451 (15%) graduates were working rurally, with 90/357 (25%) working in the same rural region as where they did rural training. Similarly, 41/170 (24%) were working in the same region as where they completed schooling. Longer duration (18–24 vs 12 months) of rural training (relative risk ratio, RRR, 3.37, 1.89–5.98) and completing both schooling and training in the same rural region (RRR: 4.47, 2.14–9.36) were associated with returning to practice in the same rural region after training.

**Conclusions:**

Medical graduates practising rurally in their early career (1–9 years post-graduation) are likely to have previous connections to the region, through either their basic medical training, their secondary schooling, or both. Social accountability of medical schools and rural medical workforce outcomes could be improved by policies that enable preferential selection and training of prospective medical students from rural regions that need more doctors, and further enhanced by longer duration of within-region training.

## Background

Rural areas continue to struggle with medical workforce shortages and poorer retention [1]. Distribution of doctors into rural regions remains problematic despite, as is the case in Australia, a growing history of government policy interventions [2]. Many determinants of rural workforce supply are now known but difficult to remedy quickly, outside of the success of recruiting or obliging immigrants to work in ‘hard to service’ areas [3, 4]. Increasingly, solutions are needed for specific regions or communities that ensure greater medical workforce self-sufficiency. Ensuring that the geographical distribution of doctors meets the health needs of populations living in rural sub-regions is a key strategy of the World Health Organization [5] and has important ramifications for achieving the health-related aspects of the United Nations’ Sustainable Development Goals [6, 7].

Evidence is building of what government policies and programs are needed to improve rural workforce distribution. Firstly, doctors with a childhood rural-origin are known to have a stronger propensity to work in rural areas compared to those with a childhood metropolitan-origin; thus, policies that increase selection of such students into medical schools are vital [1, 8, 9]. Secondly, evidence of the independent contribution of rural medical training is strengthening, both during the basic medical (undergraduate) and post-graduate training stages; thus, such pathways continue to be developed and expanded internationally [4, 10–14]. Furthermore, some medical schools now employ a social accountability mandate, with public investments in training doctors linked with a goal of returning doctors for a region [15–17]. However, there remains limited evidence about the extent to which domestically trained doctors are *returning to the same rural region(s)* where they have spent time training as a medical student (or during their earlier schooling) or, instead, whether they are choosing other work locations.

*Return to region* can be defined in many ways. At the finite level, it suggests practising in the same community as where training occurred or to where they previously resided or were schooled. This definition may be overly restrictive, however, as basic medical training commonly occurs in a range of within-region towns (delimited as suitable training posts for the curriculum to be delivered), and junior doctor residency/vocational training locations are similarly structured [9, 18, 19]. At a broader level, it may mean returning to the same region (e.g. county) or state/province, the same ‘type’ of community (e.g. small rural) or a community with similar characteristics (e.g. low socio-economic status). This approach is more useful and allows for different regionally based job and training opportunities, beyond any one town. However, most available evidence on return rates has only measured the association with similar types of areas, but not specific regions [20, 21]. The propensity for medical graduates to practice in specific higher need areas or in underserved or workforce shortage areas has also been demonstrated, but again without measuring the strength of connection between specific geographic regions where they came from and trained [22–25].

Return to region depends on a number of push and pull factors. In terms of ‘push’, it may be enhanced by repeated, longer exposure to a particular region and specific student characteristics like region of origin. Equally, as a ‘pull’ factor, it may depend, among other things, on opportunities to find work and pursue post-graduate training in the region.

Longer training periods in small rural communities can build higher levels of confidence and competence to practice in similar types of areas, though rates of return to these regions are not known [26, 27]. Many large states and regions with dispersed rural populations rely on training outposts, of various durations, as a viable avenue for attracting doctors to their community. Where wholly rural medical programs have been introduced, strong associations have been shown between recruiting and training same-state ‘locals’ and thus observing them practice in the same state (largely in rural areas) such as WWAMI’s (representing Washington, Wyoming, Alaska, Montana and Idaho) Targeted Rural Underserved Track program and Queensland’s James Cook University [28–31].

The opportunities to continue work and pursue post-graduate training in different regions vary worldwide. Some programs, including the Northern Ontario School of Medicine, provide a continuous regionally based medical training pathway through to completing medical specialisation. It has successfully built a medical workforce to match the needs of the broad region, but return rates to specific locations are not known [20]. In the United States of America, rural post-graduate medical training opportunities tend to be more limited, which is considered to associate with poorer rural supply [32], though one rural program found 60% of their graduates practising within 90 miles of their childhood hometown [33]. Australia bases 50% of its general practice training wholly in rural areas, with moderate numbers remaining in the same region where they completed this training for up to 5 years [10]. However, rural training options are often limited for junior doctors pursuing other specialties.

Apart from training experiences, Hancock et al. [34] found desire for ‘familiarity’ and ‘sense of place’ were two reasons why doctors are drawn to specific rural towns and remain there, whilst Cutchin described forming ‘habits’ in the process of place integration as key to engaging with specific rural locations [35]. Furthermore, during rural childhood, schooling or training, regional-specific skills and social and professional networks are formed, though Buttner et al. [36] identify little supporting evidence that University of Western Australia medical graduates return to their childhood rural region to work. Instead, a stronger association exists between training in a region and subsequently practising in that or a similar region.

This study aims to investigate the rate at which medical graduates with 12 months or more of rural training in a region return to practice in that same region in their early medical career. A secondary aim is whether there is an independent or additional association with their region of secondary school exposure and subsequent working location.

## Methods

### Study sample

The study is part of a large longitudinal medical school tracking study in Monash University, Victoria, Australia. The state of Victoria has over 6 million residents, with around 24% distributed rurally and Monash enrolls over 300 medical students annually through either direct (5 year) or graduate entry (4 year) pathways, converging for the final 3 years of clinical training (termed years 3 to 5 in this paper). Eligible participants commenced their medical degree after 2004 and began medical practice between 2008 and 2016. They were 1–9 years post-graduation (YPG) when outcomes were measured in 2017. This is considered ‘early career’ and is a period when doctors in Australia often work as hospital interns/medical officers, commence specialty training (typically 3–6 years duration, eligible by their second to third year after graduating), or complete their qualifications as a medical specialist (through specialist college providers, not universities). Australia’s junior doctors are active members of the workforce immediately post-graduation, competing in an open job market until enrolled in a specialist college, with those enrolled being somewhat more constrained in their training post location, depending on their specialty.

### Regional training exposure of medical students

**Fig. 1:**
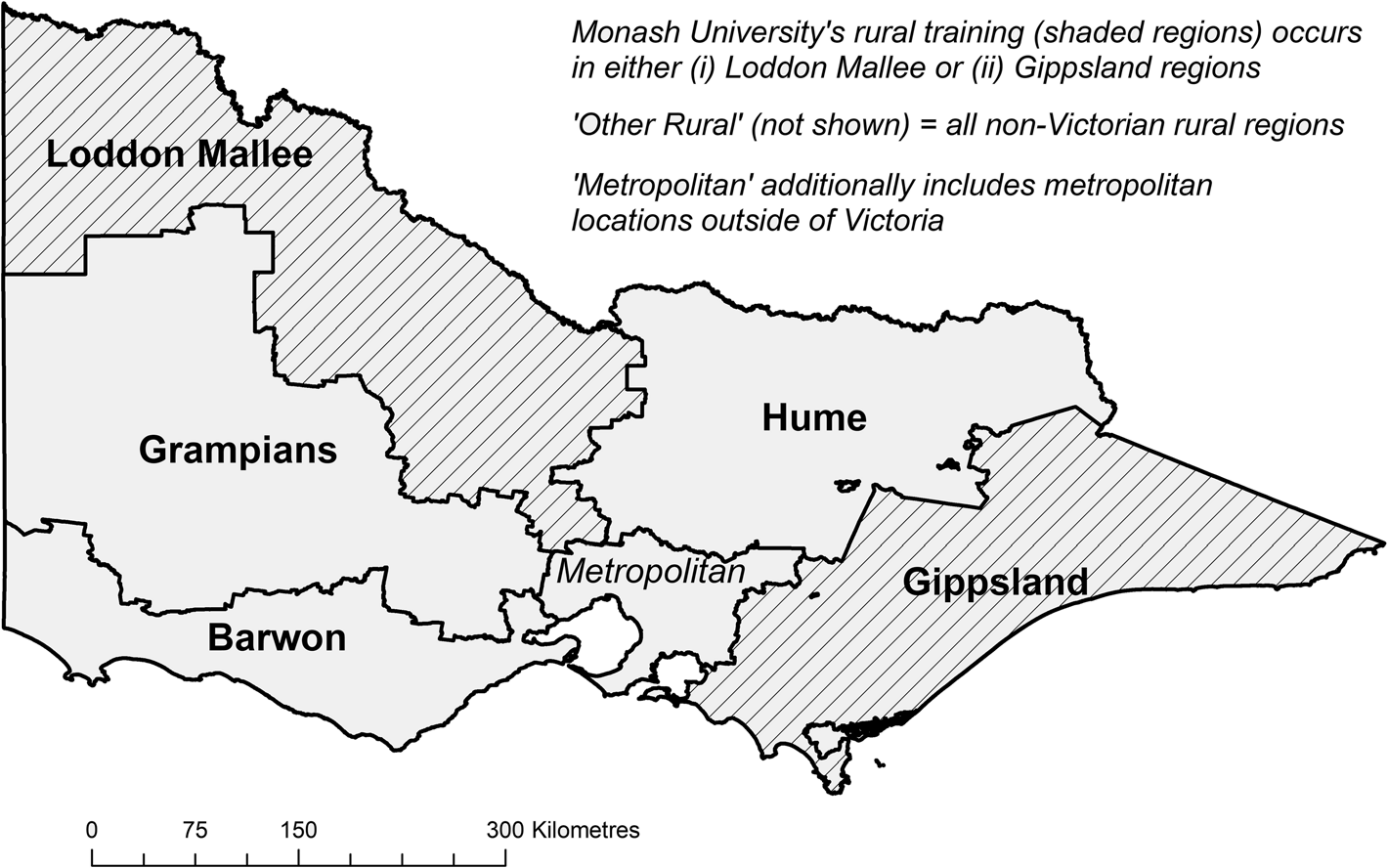
Aggregate regions used in this study for Victoria (five rural regions are shown)

Years 1 and 2 were predominantly classroom-based learning and were excluded from this analysis as they were not relatable to clinical immersion experience in a place. Information about all the locations of student basic medical training in the clinical training years, years 3 and 4 (hereafter termed ‘training’) and the duration of training in these locations was prospectively collected using university administrative data and verified by coordinators. Location of training was geocoded using Australia’s Modified Monash Model (MMM) classification, which defines rural as MMM 2–7 (MMM-2: > 50 000 population; MMM-3: 15–50 000; MMM-4: 5–15 000; MMM-5: < 5 000; MMM-6–7: remote and very remote) [37]. Duration of training in years 3 and 4 was calculated by aggregating all such periods (possible outcomes: 0, 6, 12, 18, 24 months). Year 5 clinical training (basic medical training) was excluded as it consists of 6-week core clinical rotations, which are not considered sufficient time to establish strong connections to a location. Region of training was categorised into the five main rural regional boundaries used for workforce planning in Victoria (Fig. 1). Within these, the two regions where Monash rural training occurred are shaded, namely Gippsland and Loddon Mallee.

### Student characteristics

Rural origin was defined as having resided for at least 5 years in a rural area (MMM 2–7) since commencing primary school, self-identified by a statutory declaration submitted by each student upon enrolment into medical school.

Location where secondary schooling (aged 12–18 years) was completed, geocoded according to the regions in Fig. 1, was obtained from the Medical Student Outcomes Database (MSOD) [38]. Students for whom location of secondary schooling data were missing (because they had not completed the MSOD questionnaire) were coded as ‘unknown’ location of secondary schooling, to retain these observations in the regression analysis. Data about self-reported interest in working in a rural area (measured when commencing medical school) was also obtained from MSOD. These data were available for the 2006–2014 commencing cohorts, with rural interest categorised ‘yes’, ‘no’ or ‘unknown’ to avoid dropping unmatched students from multivariate analyses.

Other relevant covariates included sex, years post-graduation (YPG), entry pathway (direct-from-school or graduate course entry) and being a recipient of either a Bonded Medical Place or Medical Rural Bonded Scholarship, both of which have a return-of-service obligation after graduation. International students at Australian medical schools were excluded from calculations requiring secondary school location.

### Outcome measure

The outcome of interest was the geographical region in which the graduate was practising (Fig. 1). This was identified using the graduate’s main work location, obtained from the national workforce registration body (Australian Health Practitioner Regulation Agency) for 2017.

A small number of graduates, who worked in another state but in a twin-city of a Victorian town, were categorised as located in the adjoining Victorian region. Otherwise, graduates with a rural main work location not in Victoria were categorised as working in ‘Other Rural’ regions. Graduates with a non-rural main work location, whether in Victoria or another state or territory, were categorised as ‘Metropolitan’.

### Analyses

Firstly, medical graduates working in 2017 were identified as being located in (i) either of Monash University’s rural training regions, (ii) a different rural region and (iii) a metropolitan region. Then, for each graduate, their work location was compared, in turn, to (1) the region where they did their training, (2) the region where they did their secondary schooling, and (3) the region/s where they did both secondary school and training. These analyses produce observed proportions only.

Secondly, a multinomial logistic regression model measured factors associated with returning to work in either the same region, a different rural region or a metropolitan area, for students who had training for at least 12 months rurally in a region. The model adjusted for key student characteristics (as described above) and used listwise deletion. Additionally, rural training duration was differentiated into two categories (12 months or 18–24 months), and a binary variable indicated whether participants had undertaken rural training in the same region as where they completed secondary schooling. Thirdly, the distribution of Monash University’s intern doctors (first year post-graduation) by region was assessed using the chi-square test by comparing it to the distribution of intern positions allocated in Victoria, the latter being strictly controlled by the Victorian Government [39]. All analyses used Stata SE 15.1 for Windows (Stata Corp, College Station, Texas) and *α* = 0.05 for statistical significance.

## Results

**Fig. 2:**
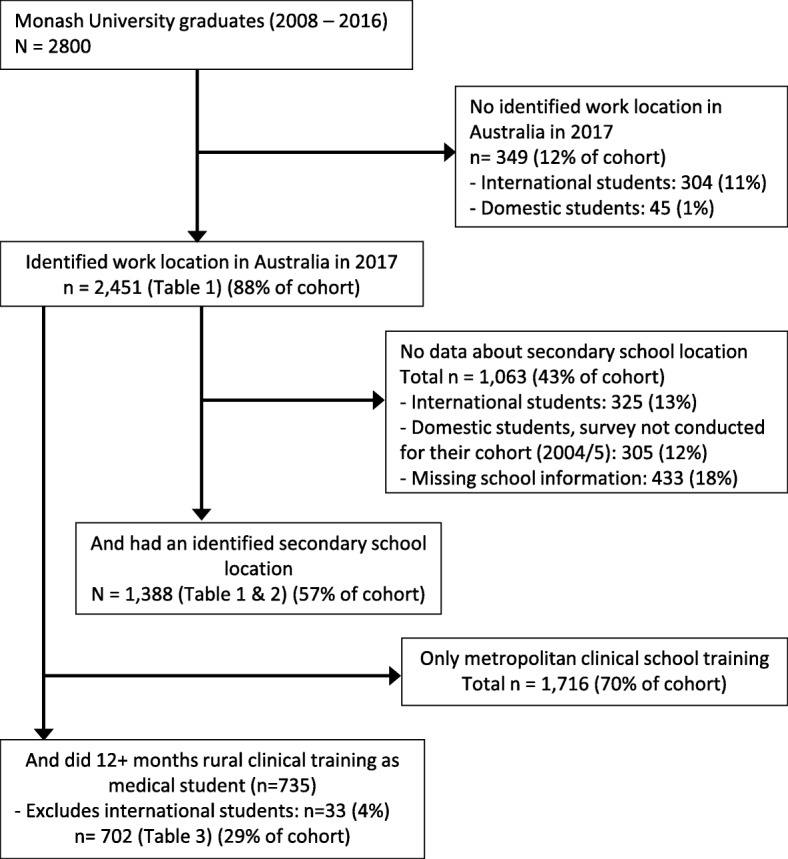
Flow chart of available work location data for Monash University graduates, 2008–2016

Figure 2 provides a flow chart of the number of observations available for each analysis. Of Monash University’s 2800 graduates in the study period, 2451 were observed working in 2017 with those missing mostly being international students. The number of observations reduced from 2451 to 1388 (43% attrition) with the addition of school location. Apart from international students being removed, YPG distribution was the main difference of observable cohort characteristics (Table 1). Secondly, the number of observations, when restricted to those having done at least 12 months training in a rural region, reduced to 702. A significantly increased proportion of this sub-group had a rural background.

**Table 1:** Cohort characteristics of Monash University graduates available for this study

		Graduates with a work location	Graduates with both work and school locations	Graduates with a work location, RCS training#
Factor	Level	*N* = 2 451	*N* = 1 388	*N* = 702
YPG	1–2	741 (30%)	538 (39%)	199 (28%)
YPG	3–4	633 (26%)	430 (31%)	171 (24%)
YPG	5–6	562 (23%)	344 (25%)	182 (26%)
YPG	7+	515 (21%)	76 (6%)	150 (21%)
Rural preference	No	1 226 (50%)	1 005 (72%)	247 (35%)
Rural preference	Yes	347 (14%)	304 (22%)	194 (28%)
Rural preference	Unknown	870 (36%)	79 (6%)	261 (37%)
Rural bonded	No	1944 (79%)	1 025 (74%)	517 (74%)
Rural bonded	Yes	507 (21%)	363 (26%)	185 (26%)
Graduate entry	No	1998 (83%)	1 126 (81%)	490 (70%)
Graduate entry	Yes	414 (17%)	262 (19%)	212 (30%)
Gender	Male	1 120 (46%)	624 (45%)	288 (41%)
Gender	Female	1 330 (54%)	763 (55%)	414 (59%)
Rural origin	No	1928 (79%)	1 052 (76%)	394 (56%)
Rural origin	Yes	523 (21%)	336 (24%)	308 (44%)
International	No	2 126 (87%)	1 388 (100%)	702 (100%)
International	Yes	325 (13%)	0 (0%)	0 (0%)
Training	Metropolitan only	1 716 (70%)	961 (69%)	Not applicable
Training	Rural: 12 months	404 (16%)	234 (17%)	377 (54%)
Training	Rural: 18–24 months	331 (14%)	193 (14%)	325 (46%)
Secondary school	Metropolitan	1 082 (44%)	1 031 (74%)	232 (33%)
Secondary school	Rural	363 (15%)	357 (26%)	195 (28%)
Secondary school	Unknown	1 006 (41%)	0 (0%)	275 (39%)

#RCS training—Monash University students trained rurally in the Loddon Mallee or Gippsland regions for at least 12 months in year 3 or 4 of the course (see Fig. 1)

YPG = (number of) years post-graduation; Rural bonded = students enrolled under the Bonded Medical Places or Medical Rural Bonded Scholarships policies; Graduate entry = pathway for 21% of Monash University students from its commencement in 2008

There were 357 (15%) observed graduates working in rural Australia (Table 2). Of these, 90 (25%) were working in the same rural regions as where they previously did rural training, 94 (26%) had trained rurally but in a different region to where they were currently working and 173 (48%) had not previously trained for at least 12 months rurally. Similar results were observed when considering secondary school location, with 42/170 (25%) working in the same region as where they completed schooling, 55 (32%) working in a different rural region to where they completed schooling, while 73 (43%) of those working rurally completed schooling in a metropolitan region.

**Table 2:** Proportions of medical graduates working in various regions^a^ in 2017 when 1–9 years post-graduation and (1) region of medical school training (years 3 and 4 of the course) (*n* = 2 451) and (2) region of secondary schooling (*n* = 1 388)

Work region^a^: 2017	(1) Medical school training location^a^				(2) Secondary school location^b^			
	Region where working 2017 (*n*)	Same rural training & work region %	Different rural training & work region %	Metropolitan training only %	Region where working 2017 (*n*)	Same rural secondary school & work region %	Different rural secondary school & work region %	Metropolitan secondary school %
Loddon Mallee or Gippsland (rural regions)	154	90 (58%)	15 (10%)	49 (32%)	94	28 (30%)	28 (30%)	38 (40%)
Other rural region	203	0 (0%)	79 (39%)	124 (61%)	76	14 (18%)	27 (36%)	35 (46%)
Metropolitan	2094	n/a	551 (26%)	1 543 (74%)	1 218	n/a	247 (20%)	971 (80%)

*n/a* not applicable

^a^Work regions are shown in Fig. 1; ‘Metropolitan’ additionally includes metropolitan locations outside of Victoria; ‘Other rural’ includes all non-Victorian rural locations

^b^MSOD (schooling) data were not available for 2004 and 2005 commencement cohorts (15%) and international students (13%). Overall, MSOD was missing for 43% of our cohort (see Table 1)

Associations between current work location and the combination of region of training and secondary schooling are shown in Table 3. Of the 94 graduates working in Monash University’s two rural training regions, 23 (24%) completed both their secondary schooling and training in the region where they were working. A further 38 (40%) completed either schooling or training in that region and 19 (20%) had either training or schooling (or both) in a rural region(s) different to where they were working, while only 14 (15%) had neither training or schooling in a rural area. Of the 76 practising in other Victorian rural regions, 20 (26%) had done their secondary schooling there.

**Table 3:** Proportions of medical graduates working in various regions^a^ in 2017 when 1–9 years post-graduation and combined regions of medical school training (years 3 and 4 of the course) and secondary schooling^b^ (*n* = 1 388)

Work region: 2017	Region where working 2017 (*n*)	Same rural region where trained and schooled	Either same rural region where trained or same rural region as schooled	Different rural region where trained and/or schooled	Metropolitan only training and schooling
Loddon Mallee or Gippsland (rural regions)	94	23 (24%)	38 (40%)	19 (20%)	14 (15%)
Other rural region	76	n/a	20 (26%)	31 (41%)	25 (33%)
Metropolitan region	1 218	n/a	n/a	460 (38%)	758 (62%)

*n/a* not applicable

^a^Work regions are shown in Fig. 1; ‘Other rural’ includes both Victorian and non-Victorian rural locations; ‘Metropolitan’ additionally includes metropolitan locations outside of Victoria

^b^MSOD (schooling) data were not available for 2004 and 2005 commencement cohorts (15%) and international students (13%). Overall, MSOD was missing for 43% of our cohort

In Table 4, key significant predictors of returning to work in the same rural region (for students who did at least 12 months of rural medical training) versus metropolitan work were having also attended secondary school in the same region as the training (relative risk ratio (RRR) 4.47, 2.14–9.36) and having a longer period of training (18–24 months compared with 12 months) in that region (RRR 3.37, 1.89–5.98). Being rural origin was also significant, but other demographic variables such as gender, being rural bonded or having an initial interest in working in a rural area were not associated with return to work in the same region as medical training occurred. There was a dose-effect association with post-graduate stage, with the odds of working in the same rural region consistently decreasing from 3 to 4 YPG to 5–6 YPG to 7+ YPG when compared to 1–2 YPG. In comparison, the only significant predictors of working in a different rural region to that where trained were being either rural origin or rural bonded. Extended exposure in the training region was not associated with other rural work, whilst school location without rural training was also not significantly associated with returning to work in that region (RRR 2.68, 0.87–8.27).

**Table 4:** Multinomial logistic regression predictive model of medical graduates working in same region as trained when 1–9 years post-graduation in 2017, for those who trained for 12 months or more in the Loddon Mallee or Gippsland region^a^ (*n* = 702)

Ref outcome: Metropolitan	Same rural region	Other rural region
	Relative risk ratio 95% CI	Relative risk ratio 95% CI
Ref group 1–2 YPG		
3–4 YPG	0.64 (0.34–1.22)	1.53 (0.77–3.02)
5+ YPG^b^	0.49 (0.27–0.90) *	1.21 (0.63–2.34)
Prefer rural at entry (ref = No)		
Yes	1.28 (0.61–2.69)	0.89 (0.44–1.83)
Unknown	1.81 (0.69–4.77)	0.72 (0.28–1.85)
Rural bonded	1.49 (0.85–2.59)	2.26 (1.33–3.84)**
Graduate entry	1.37 (0.79–2.38)	1.10 (0.62–1.96)
Female	1.18 (0.71–1.97)	1.33 (0.80–2.23)
Rural origin	1.89 (1.05–3.43)*	4.08 (2.26–7.36)**
Medical training in the region^a^ for 18–24 months (ref = 12 months)	3.38 (1.91–5.98)**	1.22 (0.72–2.06)
School location (ref = Schooled in other region)		
Both schooling and training (and working) in same region	4.47 (2.13–9.35)**	n/a
Both schooling and working (not training) in same region	n/a	2.62 (0.85–8.05)
Missing school information	0.91 (0.39–2.12)	2.23 (1.01–4.91)

*n/a* not applicable, *YPG* years post-graduate

*p < 0.05, **p < 0.01

^a^Work regions are shown in Fig. 1; ‘Same rural’ includes either Loddon Mallee or Gippsland regions; ‘Other rural’ includes both Victorian and non-Victorian rural locations; ‘Metropolitan’ additionally includes metropolitan locations outside of Victoria

^b^YPG 5–6 and YPG 7+ were collapsed together because few observations with school location were available for YPG 7+ (see Table 1)

Proportionally, the distribution of allocated rural intern places in Victorian regions in 2017 was Barwon—6%, Grampians—20%, Loddon-Mallee—27%, Hume—35% and Gippsland—13%. In comparison, the distribution of 1-YPG graduates from the Monash cohort in these regions in 2017 was 3%, 5%, 37%, 33% and 23% respectively. Overall, there was a significant association between training in the Loddon Mallee and Gippsland regions and working first year post-graduation in these regions (*p* = 0.002).

## Discussion

Our study provides new empirical evidence of the likely importance of medical program design for achieving doctors who return to work in particular rural regions. Notably good design includes education that is rurally distributed within a region, offers longer duration and selects students who were schooled in the same region, in order to increase local rural medical workforce supply for a particular region. Our study reports a large and statistically significant association (RRR 4.5) between both medical training and secondary schooling in a specific region of Victoria and subsequent work in that region. These findings have significant policy implications, with closer attention currently given to selection tools predicting successful course completion rather than workforce distribution [40, 41].

Selection into a medical degree in Australia is commonly unrelated to where students grew up. The US employs a range of ‘in-state’ selection policies, but recent statistics still show that around 40% of these students graduate from an interstate medical school [42]. This aspect of student selection contrasts with the social accountability mandate which has somewhat succeeded in improving rural workforce distribution in parts of Canada. Students from a specific region of the country—such as from Northern Ontario, which had experienced longstanding medical workforce shortages [43]—are preferentially selected into the region’s medical school. They report for many such communities there is the benefit of having connections and contributing to the training process through the return of doctors that they hosted as medical (or earlier schooling) students [44]. Michigan State University’s small but long-standing Rural Physician Program has also achieved similar success with Upper Peninsula origin students both training and subsequently working in that region [22]. There is a growing imperative, worldwide, that graduating doctors will meet the health care needs of the population, rather than purely addressing their professional interests [45]. These findings about the factors that promote return to rural regions may play a part in supporting community self-sufficiency for a supply of doctors.

In addition to training distribution, duration of training matters with significantly stronger return to region rates seen for students who train for 18–24 months compared with those who train for 12 months (RRR 3.4). This finding supports other evidence of longer-term rural outcomes being associated with longer rural exposure [46]. Notably, 12 months of rural exposure is the current minimum level expected in Australian policy for at least 25% of each medical school’s graduates, though consideration should be given to supporting training models that enable longer periods within a region.

Our data confirm that significantly more than is proportionally expected of Monash University’s graduates initially (first year post-graduation, 1-YPG) practice in its training regions and fewer than expected are in regions where training is delivered by other universities. However, the finding of diminishing return to region from 3-YPG through to 7+ YPG is likely to be a reflection of the early career distribution of doctors being strongly dictated by limitations in prevocational/residency (2+ YPG) and enrolled vocational training opportunities (starting point varies, mostly between 3-YPG and 6-YPG) in rural Australia. For the latter, vocational training distribution is largely dependent upon the location of accredited training posts as dictated by the medical specialist colleges with most specialty training positions, other than general practice posts, based in metropolitan locations [47]. It is possible that the Australian government’s 2017 commenced national Regional Training Hubs initiative may result in stronger regional and rural post-graduate training pathways in years to come and improve the ‘pull’ to long-term work in specific regions.

Our data additionally show that some Monash graduates working in the rural regions had neither basic medical training exposure nor completed their secondary school in the same regions where they are working. Around 35% (Monash training regions) and 74% (non-Monash training regions) of Monash graduates working in rural Victoria had no observable linkage to that region. Upon commencing employment, these graduates are less likely to have a detailed understanding of the context in which they work and fewer social and professional connections in the region compared with graduates who have either trained or attended secondary schooling in the region. Additional supports may therefore be required, especially initially, in order to optimise their regional retention. It is, however, unclear from our data whether doctors with no observable connection to the regions they were practising in did in fact have some other unobserved linkage to those regions.

A limitation of this study is that a majority of observed graduates are still at an early career stage and a small proportion may have commenced training and working as fully independent medical specialists. However, specialist training location and a generalist specialty choice remain important outcomes given the strengthening longitudinal evidence linking this with subsequent independent rural practice [10, 13, 20]. This was not possible to explore using our data, but is planned in future studies.

A further limitation is that no measure of rural connection or rural interest to specific regions, outside of where secondary school was completed was available for our cohort. It is possible that participants moved between different regions, both metropolitan and rural, during their childhood. Other connections not observable in this study due to its use of administrative datasets, such as parent’s and partner’s rural background, networks related to extended family and friends or childhood holiday areas, or even preferences for where they raise their children [48], may have strengthened connections to specific regions thus potentially explaining some of the observed distribution patterns. Additionally, our study only observes outcomes for graduates of a single university of Australia and for a single point in time. Our data do not reveal how long a doctor has been working in a region, thus it is plausible some may have previously worked rurally but be metropolitan at the time of data collection and vice versa. Despite this study’s large cohort size, the measurement of return to region was at an aggregated ‘region’ level because the program immerses students in only two distinct larger regional centers, along with time in smaller nearby communities. It was therefore not possible to assess ‘return to region’ at a more granular level.

## Conclusion

Medical graduates practising rurally in their early career (up to 9 years post-graduation) are likely to have previous connections to the region, through either their basic medical training, their secondary schooling, or both. Return rates are highest when rural training duration is longer (18–24 months) and when rural training and secondary schooling are in the same region. Improved social accountability of medical schools and better rural medical workforce outcomes could be achieved through policies that enable preferential selection of prospective medical students from rural regions experiencing medical workforce shortages and by training those students within the same region for longer periods of their basic medical training.

## Abbreviations

MMM: Modified Monash Model
MSOD: Medical Student Outcomes Database
RCS: Rural Clinical School
RRR: Relative risk ratio
WWAMI: (collaboration of) Washington, Wyoming, Alaska, Montana and Idaho
YPG: (number of) Years post-graduation
